# Ultra-Wideband Ranging Error Mitigation with Novel Channel Impulse Response Feature Parameters and Two-Step Non-Line-of-Sight Identification

**DOI:** 10.3390/s24051703

**Published:** 2024-03-06

**Authors:** Hongchao Yang, Yunjia Wang, Shenglei Xu, Jingxue Bi, Haonan Jia, Cheekiat Seow

**Affiliations:** 1 The Key Laboratory of Land Environment and Disaster Monitoring, China University of Mining and Technology, Xuzhou 221116, China; hongchao_yang@cumt.edu.cn; 2 The Navigation Institute of Jimei University, Xiamen 361021, China; jmu-xsl@jmu.edu.cn; 3 The School of Surveying and Geo-Informatics, Shandong Jianzhu University, Jinan 250101, China; bijingxue19@sdjzu.edu.cn; 4 The School of Instrument Science and Engineering, Southeast University, Nanjing 210096, China; jiahaonan1022@163.com; 5 The School of Computing Science, University of Glasgow, Glasgow G12 8RZ, UK; cheekiat.seow@glasgow.ac.uk

**Keywords:** ultra-wideband (UWB), indoor positioning and navigation, non-line of sight (NLOS), channel impulse response (CIR), ranging mitigation, deep learning

## Abstract

The effective identification and mitigation of non-line-of-sight (NLOS) ranging errors are essential for achieving high-precision positioning and navigation with ultra-wideband (UWB) technology in harsh indoor environments. In this paper, an efficient UWB ranging-error mitigation strategy that uses novel channel impulse response parameters based on the results of a two-step NLOS identification, composed of a decision tree and feedforward neural network, is proposed to realize indoor locations. NLOS ranging errors are classified into three types, and corresponding mitigation strategies and recall mechanisms are developed, which are also extended to partial line-of-sight (LOS) errors. Extensive experiments involving three obstacles (humans, walls, and glass) and two sites show an average NLOS identification accuracy of 95.05%, with LOS/NLOS recall rates of 95.72%/94.15%. The mitigated LOS errors are reduced by 50.4%, while the average improvement in the accuracy of the three types of NLOS ranging errors is 61.8%, reaching up to 76.84%. Overall, this method achieves a reduction in LOS and NLOS ranging errors of 25.19% and 69.85%, respectively, resulting in a 54.46% enhancement in positioning accuracy. This performance surpasses that of state-of-the-art techniques, such as the convolutional neural network (CNN), long short-term memory–extended Kalman filter (LSTM-EKF), least-squares–support vector machine (LS-SVM), and k-nearest neighbor (K-NN) algorithms.

## 1. Introduction

Indoor high-precision localization is a rapidly growing field, driven by the increasing demand for indoor location-based services such as emergency response, navigation systems, and the Internet of Things (IoT). However, commercial Global Satellite Navigation Systems (GNSSs) [1] are not designed for indoor location services due to severe interference from building structures. As a result, researchers have explored various techniques for indoor positioning, including fingerprint-matching methods based on Wireless Fidelity (Wi-Fi) [2,3], Bluetooth [4], or geomagnetism [5]; ranging positioning methods based on ultra-wideband (UWB) [6,7] and pseudo-satellite systems [8]; and angle-positioning methods [9] based on antenna array technology. Among them, UWB has emerged as a promising technology for accurate position estimation and synchronization control in harsh indoor environments. Its attributes of high time resolution, ability to penetrate obstructions, resistance to multipath interference, and low power consumption facilitate stable and high-precision ranging and positioning in line-of-sight (LOS) channel states. Existing UWB ranging-based positioning systems PlusON [10] and Localizer [11] can achieve centimeter-level positioning accuracy in LOS environments. With the advent of antenna array technology [12], integrating angle measurements with ranging results enables UWB single-base-station positioning, but this approach has a stronger dependency on LOS channel environments. The LOS phenomenon refers to the scenario where the direct physical path between devices is unobstructed by any barriers, whereas the non-line-of-sight (NLOS) [13] phenomenon is the opposite. In NLOS scenarios, obstacles impede the propagation of UWB signals, preventing the hardware from acquiring the true first-path (TFP) signal corresponding to the actual distance. This results in positive bias in range measurements based on time of flight (TOF) [14], thereby affecting positioning accuracy. Due to the complexity and variability of indoor environments, the frequent occurrence of NLOS phenomena restricts the application and development of UWB positioning. Therefore, precise channel identification to accurately extract LOS ranging errors and to mitigate or eliminate NLOS ranging errors is crucial for the efficacy of Indoor Positioning Systems (IPSs) that rely on UWB as their core technology.

NLOS identification algorithms consist of two parts: feature parameter extraction and classification algorithms. There are various feature parameters, including distance [15,16,17], coordinates [18,19,20], and channel impulse response (CIR) [21,22,23]. Common classification algorithms include threshold comparison [16], neural networks [24,25,26], support vector machines (SVMs) [27,28,29], and k-nearest neighbor [30]. Schroeder et al. [16] implemented a fixed threshold-based mathematical statistical analysis of UWB ranging distances for NLOS channel identification. Although simple, this method exhibits poor and unstable identification accuracy. Coordinate features are derived from redundant information or additional techniques. For instance, real-time positioning results from the Inertial Measurement Unit (IMU) can enhance UWB channel state accuracy, but the cumulative error inherent in IMUs can constrain the performance of the integrated system [20]. Sequential CIRs contain information about the channel state, including their energy and timing features, which, when combined with machine learning algorithms, can be utilized for channel identification. Kegen et al. [29] trained a least-square SVM (LS-SVM) to identify NLOS data using two inputs: maximum CIR amplitude and mean excess delay. This approach surpassed the first two in both accuracy and timeliness, yet its performance was subject to fluctuations due to external conditions (environment and obstacles). Sequential CIRs hold implicit information reflective of external conditions, making them a focal point of current research. However, a prevailing challenge is the limited variety of CIR features used and the algorithms’ insufficient exploration of latent information.

Based on the results of NLOS identification, the algorithm deletes or mitigates NLOS ranging errors to reduce the influence of the NLOS phenomenon on UWB positioning. Deleting NLOS ranging results can eliminate the impact of its error, but the algorithm needs to add UWB base stations [31] or introduce additional technologies [32,33,34] to ensure the accuracy and continuity of positioning. Chen et al. [18] used weights that were inversely proportional to the normalized residual to combine the ranging values of multiple UWB nodes to suppress NLOS errors. The algorithm requires a sufficient density of UWB anchors, and the computational complexity geometrically doubles with the increase in density. In contrast, mitigating NLOS errors [35,36,37] is a more efficient and cost-effective strategy, reducing the instability introduced by additional techniques. By pre-training the correlation between the CIR features and ranging errors, the algorithm can accurately and efficiently mitigate NLOS ranging errors. Marano et al. [21] used five CIR features in combination with LS-SVM to reduce the standard deviation (STD) of ranging errors by 30.36%. This approach enhanced the accuracy of ranging and positioning, but its performance was somewhat unstable, as it is overly reliant on the accuracy of NLOS identification and does not fully exploit the UWB signal. To address these problems, we propose a novel UWB ranging error mitigation algorithm using new CIR features and a two-step NLOS identification algorithm. The main contributions of this paper can be summarized as follows:

1. We divide the entire process of UWB signal collection into three stages based on the CIR fluctuation trend caused by UWB signal arrival: the environmental noise stage, CIR steep rise stage, and CIR slow descent stage. To the best of our knowledge, this innovative classification is the first to be utilized for both UWB NLOS identification and ranging error mitigation. Leveraging the unique characteristics of these stages, we optimize existing CIR features and propose two new CIR features from key nodes—TFP delay and energy rise—which have much stronger feature representation and robustness and are first used to cover the leading edge detection algorithm for UWB signal identification.
2. For channel identification, we propose a two-step NLOS identification algorithm that leverages a decision tree (DT) to pre-extract typical LOS and NLOS data and then uses the feedforward neural network (FNN) to fine-tune the remaining data. Moreover, we introduce fuzzy logic, i.e., the probability of a CIR feature being identified as LOS, to extract the potential information, ensure the accuracy of the DT, and optimize the initial state of the FNN. To bolster the robustness of our algorithms, we adopt a dynamic update policy for the DT threshold, which is based on the final identification results.
3. For ranging error mitigation, we propose a novel method of categorizing NLOS ranging errors into three types based on the underlying causes of the errors and their waveform characteristics. For each type of NLOS ranging error, we take first-path (FP) detection as the core to optimize the corresponding correction strategy. To fully leverage the capabilities of the UWB signal system, this study implements a recall mechanism designed to extract high-precision ranging results. Furthermore, this paper classifies and partially mitigates LOS ranging errors to further reduce the dependence of positioning performance on the accuracy of NLOS identification. Finally, we validate the performance of the newly proposed features and algorithms, as well as their enhancement in dynamic positioning accuracy, through a series of experimental activities across multiple scenarios.

The remainder of this paper is organized as follows. Section 2 briefly reviews the related works about UWB NLOS identification and ranging error mitigation. Section 3 introduces the extraction of new CIR features and the concept of fuzzy logic. The details of the two-step NLOS identification algorithm and ranging error mitigation strategy are described in Section 4. We test the performance of the algorithms through static and dynamic experiments involving humans, walls, and glass obstacles at two specific test sites in Section 5. Finally, Section 6 summarizes the work in this paper and suggests future research.

## 2. Related Works

Since the Federal Communications Commission (FCC) authorized the use of UWB for civilian applications in 2002, numerous research teams have developed a variety of positioning systems based on multi-base-station UWB ranging and intersection positioning techniques. Among these, the Localizer positioning system, utilizing the Time Difference of Arrival (TDOA) ranging algorithm, achieves a positioning accuracy of 0.05 m in 30 m LOS environments. However, the widespread occurrence of NLOS conditions indoors often introduces a positive bias in ranging measurements, which complicates the accurate pinpointing of target coordinates. To counteract this, such technologies typically involve deploying an adequate number of base stations within the positioning area, thus enhancing the proportion of LOS ranging results. With the advent of antenna array technology, these systems have evolved to simultaneously capture both the distance and angle between the target point and known reference points, facilitating single-base-station positioning. This approach significantly reduces the number of devices required, but it comes with higher hardware costs and increased complexity in signal processing. Furthermore, the NLOS phenomenon adversely affects the precision of both range and angle measurements in these technologies, diminishing the system’s fault tolerance and heightening its reliance on LOS channel conditions. Consequently, the essence of UWB positioning algorithms is rooted in accurately discerning high-precision LOS results through channel identification algorithms and mitigating the less precise NLOS results, thereby ensuring the reliability and accuracy of the overall positioning output.

### 2.1. UWB NLOS Identification

Existing wireless NLOS identification algorithms [3,15] are broadly classified into three categories based on the nature of their feature parameters. Distance-based methods utilize the difference in variance or probability density functions [15] of ranging distances in diverse environments for channel state identification. The threshold [16] for NLOS identification is derived from repeated prior data. However, they are characterized by low accuracy and robustness, with their effectiveness significantly diminishing in varying motion states. Coordinate-based methods ascertain NLOS channel states using positioning data derived from UWB ranging distances or ancillary techniques. The authors of [18,19] utilized the coordinate discrepancies of multiple transition points, obtained from assorted ranging results, to detect NLOS data when the anchor density is sufficient. Although these methods do not necessitate additional technology, their accuracy is inversely proportional to the increase in NLOS data. In systems where IMUs and UWB are tightly integrated, the channel state is determined by the real-time location of the IMU [20,38]. Although this method does not require the modeling of the channel environment, it is limited by the cumulative error of the IMU and the inability to pinpoint the source of NLOS errors. A hybrid strategy that replaces IMUs with high-precision binocular vision [39] has also been used, which can obtain indoor maps and provide better performance. However, the system is complex and not portable. Furthermore, the limitations associated with the additional technology restrict the universality and accuracy stability of the algorithms.

CIR-based methods utilize features from the CIR sequence, which encapsulates information about the environmental topology and obstacles. The convolutional neural network (CNN) employed in [24] used the CIR raw data to identify NLOS errors, achieving an average accuracy of 90% across various architectures, including ResNet and Encoder. Despite its high sensitivity to extreme NLOS scenarios, this approach is time-intensive due to redundant information [25]. To address this issue, Jiang et al. [26] introduced a long short-term memory (LSTM) layer in place of the fully connected layer, and the application of reversible transformations to de-noised CIR data effectively simplified the network structure [40]. Nonetheless, the improvement in NLOS identification accuracy was marginal. Moreover, this method, focusing primarily on the timing aspects of the CIR sequence, tends to overlook the signal’s energy attenuation. Existing manually extracted CIR features derived from CIR sequences include energy features, which are based on obstacle energy attenuation (total CIR energy, first-path power level, receive power level) and timing features of multipath delay components (kurtosis, mean excess delay, root-mean-square delay) [21,22]. When employing the same structural SVM for NLOS identification, the average LOS and NLOS recall rates for a single CIR feature are 70.54% and 72.02%, respectively [27]. This low LOS recall rate adversely affects the preservation of high-precision ranging results, with the maximum accuracy gap between parameters reaching approximately 20%. Under varying external conditions, the LOS recall rate for CIR feature kurtosis decreased by 6.3%, whereas the NLOS recall rate increased by 1.18% [28]. Cwalina et al. [23] developed a feedforward neural network (FNN) using two inputs—total CIR energy and first-path power level—achieving an accuracy of at least 90.1% in mixed-channel environments. However, this algorithm only considers humans as obstacles and operates in simple tracks. Guvenc et al. [28] compared the joint likelihood ratio constructed by the probability density function of kurtosis, mean excess delay, and root-mean-square delay with a fixed threshold to identify NLOS data. This approach improved the mean LOS/NLOS recall by 6.78%/5.7% compared to a single CIR feature, but it lacked mixed-channel experiments. Heidari et al. [36] introduced a novel CIR feature for detecting the first-path signal using a hybrid approach, consisting of the first-path power level and TOF, and established a joint likelihood ratio based on the mean excess delay and total CIR energy to further categorize NLOS into undetected and detected direct paths. However, its threshold is not well-defined and requires recalibration with changes in external conditions. Marano et al. [21] explored the impact of different compositions of the CIR feature vector ($V_{CP}$) on SVM performance. Increasing the number of CIR features in $V_{CP}$ can cause fluctuations in the algorithm’s identification performance, as noted in [29]. The accuracy in [29] was 3% higher than that in [28] under the same $V_{CP}$ conditions. Using the same three CIR features in $V_{CP}$, Kolakowski et al. [22] substituted the “mean excess delay” and “root-mean-square delay” with “the difference between the receive power level and first-path power level” and “total CIR energy”, enhancing the LOS recall by 51.35% and improving identification accuracy to 93.67%. Clearly, optimizing the structure of $V_{CP}$ for different external conditions is crucial to maximize SVM performance. On one hand, the current CIR feature types, namely timing and energy features, fail to adequately distinguish key communication nodes and stages across various channels. These features are not integrated with signal detection algorithms, and their scope is confined to differentiating between multipath signals, without considering environmental noise. On the other hand, identification algorithms solely use the value of the CIR feature as the input vector, neglecting its potential information and failing to optimize models.

### 2.2. NLOS Ranging Suppression

Based on whether NLOS ranging errors are deleted or mitigated, this paper classifies existing NLOS ranging suppression algorithms into two primary methods: fusion and mitigation. The fusion method aims to suppress NLOS ranging errors by incorporating additional data into the positioning calculation [31,41]. Venkatesh et al. [31] used LOS ranging to estimate the objective function and NLOS ranging to restrict the feasible region in linear programming. This method outperformed the least-squares approach in mixed-channel environments but proved ineffective in exclusively NLOS conditions. Based on weight [18], Jiao et al. [19] further achieved an average 58% reduction in computation time by selecting combinations with minimal residual error. However, optimizing the anchor layout is crucial to minimize the proportion of NLOS data and ensure algorithmic efficiency. Yao et al. [32] combined the IMU with UWB to mitigate NLOS errors, enhancing both the continuity of the positioning track and the accuracy, which was 2.5 to 5 times greater than that of the least-squares and trilateration algorithms. However, the system performs poorly in continuous NLOS environments. Alternatively, a binocular camera can replace the IMU, eliminating cumulative errors and improving positioning accuracy by 27% on the *Y*-axis [33]. Although this system smoothens the track and reduces visual positioning drift, it compromises portability and increases computational demands. Zheng et al. [34] employed IMU-assisted visual position matching and integrated the discrepancy between visual positioning and UWB location results through filter fusion to derive the final outcome, enhancing the average positioning accuracy by over 20%. However, this method does not fully exploit IMU data. Fusion methods, bypassing the need for error modeling or extensive prior data from repeated measurements, are constrained in their ability to consistently suppress errors due to environmental limitations inherent in auxiliary techniques. The mitigation methods of NLOS ranging errors require the establishment of a mapping model between the ranging errors and their features. These errors primarily arise from the system’s inaccurate detection of the TFP in UWB signals, leading to an overestimation of the TOF and ranging results. Consequently, such algorithms are often referred to as FP detection methods. Wu et al. [35] employed maximum likelihood estimation, combining TOF and a CIR feature (maximum CIR amplitude), to deduce the TFP. They formulated the NLOS ranging error expression based on the signal-path loss model, reducing the mean ranging error from within 10 m to less than 0.5 m. However, this approach incurs a delay due to iterative estimation. The optimal composition of the CIR feature vector ($V_{CP}$) varies under different external conditions. For instance, the performance of the $V_{CP}$ with five CIR features in [21] was 0.019 m less accurate than the $V_{CP}$ (maximum CIR amplitude, mean excess delay) in [29]. Heidari et al. [36] enhanced pulse waveform identification for different path signals by preprocessing the CIR with a finite bandwidth filter, thereby improving ranging accuracy. However, this method is not time-efficient during post-processing. Wang et al. [37] developed a generalized maximum likelihood approach based on the assumption of strong path synchronization at the receiver, reducing the TFP search space. Incorporating timestamp and amplitude threshold information [42] further narrowed the search area by 30%. Nonetheless, this method is vulnerable to random non-Gaussian outliers with high energy. Song et al. [43] converted the signal amplitude into statistical rank information to suppress heavy-tailed non-Gaussian noise or outliers while effectively preserving TFP data. The algorithm re-evaluates the TFP by setting a threshold, combining the row-rank statistical sequence of the signal frame, and integrating a multipath signal detection algorithm to enhance performance. Although it corrects amplitude outliers more effectively than CIR averaging, it requires more time to traverse all paths and additional signal collector hardware. Li et al. [44] used the least-square method to locate the TFP, ensuring that 80% of the ranging error was less than 1 m. This method sacrifices mitigation performance but significantly reduces complexity. Mitigation methods are generally stable and unaffected by environmental variables, yet they are complex and fail to balance performance with timeliness. Moreover, these algorithms do not adequately mitigate LOS data or fully exploit UWB’s anti-multipath and obstacle penetration capabilities, leading to low data utilization and high dependency on the precision of the identification algorithm.

## 3. Theoretical Framework

### 3.1. UWB Channel CIR Feature Extraction

**Figure 1:**
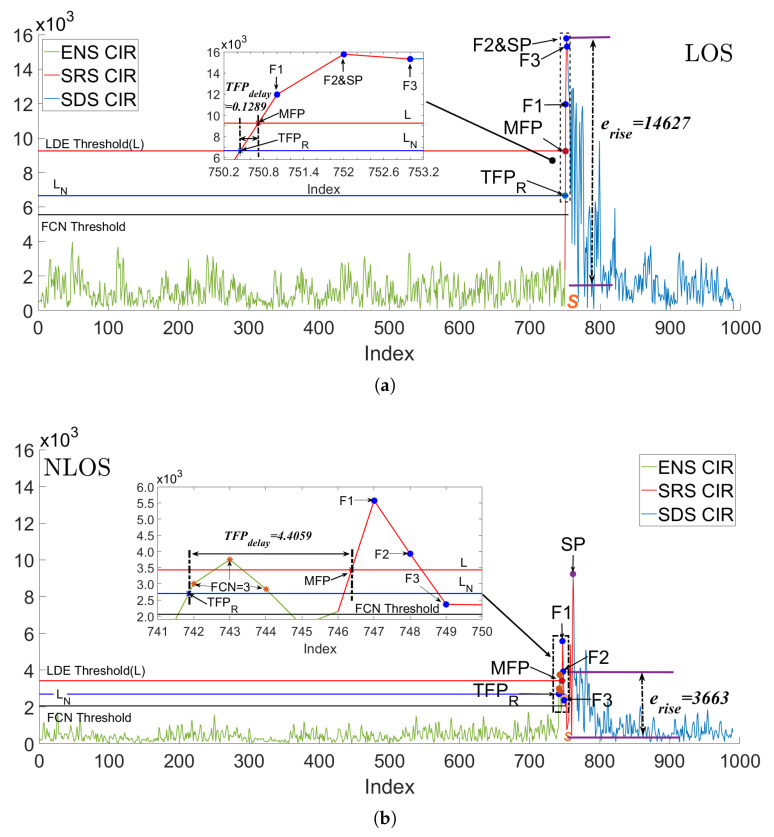
Typical CIR fluctuations and some key nodes in the three communication stages of UWB (ENS, SRS, and SDS) under different channel environments, and depiction of the LDE algorithm and new CIR features on the CIR waveform: (**a**) LOS, (**b**) NLOS.

UWB technology estimates distances by calculating the time of flight of signals between devices. In the ranging process, the UWB transmitter emits signals at a predetermined frequency and logs the transmission time. Concurrently, the UWB receiver continuously monitors the received signal’s energy level. When this level surpasses a predefined threshold set by the built-in leading-edge detection (LDE) algorithm, the corresponding time is marked as the signal arrival time. The system then computes the ToF using both the signal emission and arrival times, which is pivotal for accurate ranging results. The LDE algorithm plays a critical role in this process by determining the threshold and the precise signal arrival time based on the CIR. The CIR is a digital measurement of the energy of the received UWB signal and environmental signal noise by UWB hardware. Within the unit time (1.0016 ns) of a UWB quartz clock, the hardware estimates the correlation between the cumulative input sample and the expected potential customer sequence to calculate the CIR, which is recorded as r(t) [26,45,46]. Figure 1a shows the major effective CIRs during one communication in typical LOS. The hardware uses the threshold (*L*) from the embedded LDE algorithm [47] to identify the FP, and its formula is as follows:

(1) L=S∗NTM,

where *S* is the standard deviation of the CIRs, indicative of the environmental noise level, and NTM is the noise threshold multiplier, which is set to a constant value of 13 [48].

As illustrated in Figure 1, the UWB system continuously evaluates the CIR, while the LDE algorithm dynamically calculates the environmental noise level *S* (represented by the purple horizontal line) and updates the FP judgment threshold *L* (depicted as the red horizontal line) in real time based on the formula. When the CIR first surpasses the dynamic threshold *L*, the system records this moment as the arrival time of the Measured First Path (MFP), as shown in Figure 1. This study categorizes the CIRs (green line in Figure 1) preceding the MFP, as reported by the LDE, as the environmental noise stage (ENS). These represent signal noises from the environment and, according to the LOS theory, do not contain UWB signals. Points F1, F2, and F3 in Figure 1 correspond to the CIR amplitudes at three distinct moments post-MFP, closely associated with the signal energy. UWB signals, emitted from the transmitter antenna, reach the receiver through various paths, causing a gradual increase in the CIR. In indoor environments, where the dimensions are relatively small compared to the speed of light, some signals may arrive within intervals shorter than the quartz clock’s resolution, resulting in their recording as a superposition. This leads to the formation of a single CIR with maximum amplitude, identified as the Strongest Path (SP) in Figure 1, marking the culmination of the CIR rise process due to the UWB signal. As depicted in Figure 1a, this paper classifies the CIRs (red line in Figure 1) between the MFP and SP as the CIR steep rise stage (SRS). The subsequent CIRs (blue line in Figure 1) comprise multipath (MP) signals, whose energy progressively diminishes with increased transmission distance, defining the CIR as the slow descent stage (SDS).

In the NLOS scenario depicted in Figure 1b, the TFP signal is attenuated by obstacles and obscured within the ENS, with its amplitude falling below the dynamic threshold *L* set by the LDE algorithm, leading to the misidentification of the TFP. The contrast between the NLOS (Figure 1b) and LOS (Figure 1a) conditions has prompted researchers to develop numerous CIR features to differentiate between channel states. However, the reliance on a substantial portion of the CIRs introduces certain limitations to the existing CIR features. Firstly, processing a large volume of CIRs generates redundant information, which can result in the underutilization of the identification capabilities of CIR features and increased latency. Secondly, the current CIR features, which are independent of the LDE and critical communication stages, are unidimensional, focusing solely on the mathematical characteristics of the entire sequence of CIRs, thereby compromising environmental robustness. To address these shortcomings, this paper introduces two novel parameters: TFP delay ($TFP_{delay}$) and energy rise ($e_{rise}$), which are formulated in light of the limitations of the LDE and the distinctions between the TFP signal and signal noise levels.

(1) TFP delay ($TFP_{delay}$)

The LDE algorithm dynamically evaluates the signal noise level using the parameter *S* in (1). However, as shown in Figure 1, *S* (purple horizontal line) alone cannot effectively distinguish signal noise from the TFP for threshold comparison, leading to misjudgment of the TFP caused by the fluctuation and mutation of signal noise. To address this, the LDE algorithm introduces NTM to effectively distinguish between signal noise and the TFP, thus preventing misjudgment. However, the fixed NTM is unsuitable for the variable external environment, which may result in the incorrect classification of certain UWB signal CIRs as *S*, thereby further enhancing *L*. This creates a vicious cycle and significant delay between the MFP and TFP. To address this issue, we initially organize the CIRs in the ENS in ascending order and construct the Cumulative Distribution Function (CDF). The inverse function of the CDF, denoted as $CDF_{ENS}^{-1}\left(0.9\right)$, is utilized to establish a new signal noise level. The ‘−1’ in the upper right corner signifies the inverse function. Under four experimental campaign scenarios, starting with 50% of the CIRs as the baseline for environmental noise and incrementally increasing this proportion, we observe that the ratio of the average values of the remaining and utilized CIRs predominantly ranges between 2.5 and 3.5. As the proportion escalates from 80% to 86%, it stabilizes approximately between 2.91 and 2.92. Continuing this increase, the ratio reaches an average of 2.98 at 90%, with a growth rate of 0.01. Beyond this point, further escalation in the CIR ratio results in an increased growth rate of 0.02 to 0.05. This indicates that the amplitude of the residual CIRs in the sequence is significantly higher than that of the earlier CIRs, suggesting their origin from either the UWB CIR or anomalous signal noise. Consequently, to accurately gauge the real environmental noise, this study considers 90% of the CIR as representative of environmental noise. Additionally, a new multiplier similar (=3) to NTM is chosen to offset the signal noise mutation. The value 3 was chosen based on the four experimental campaign scenarios that were each repeated 20 times to give rise to the highest LOS/NLOS differentiation with a $TFP_{delay}$ accuracy of around 60% to 73%. Consequently, the new threshold ($L_{N}$) used to identify the UWB signal is given by:

(2) $$L_{N}=CDF_{ENS}^{-1}\left(0.9\right)*3,$$

**Figure 2:**
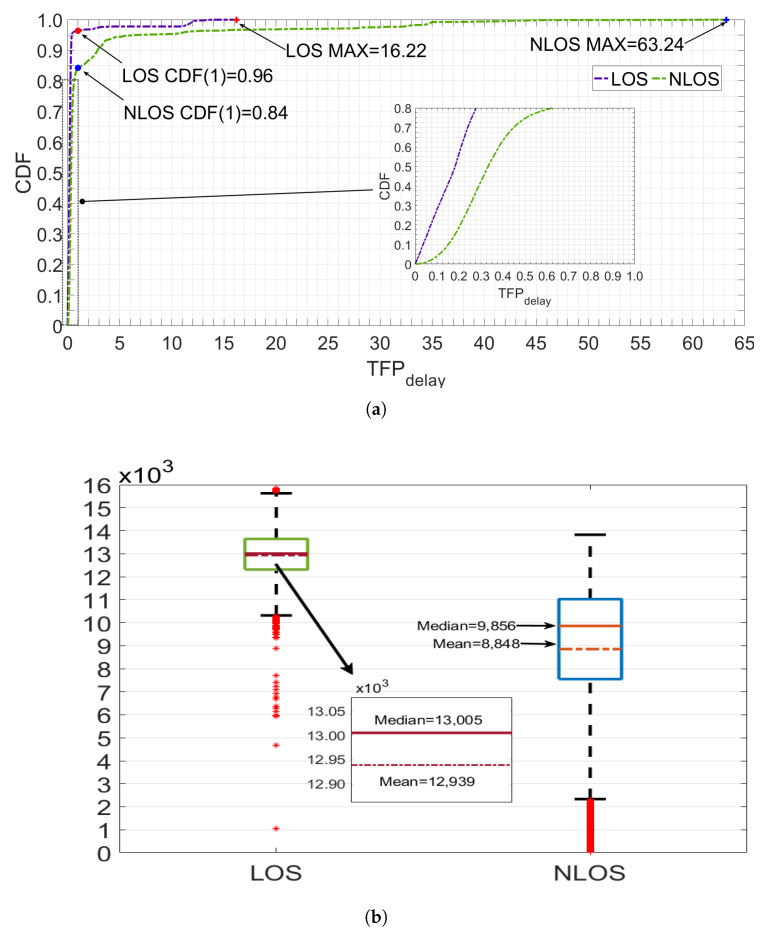
The data distributions of the new parameters: (**a**) $TFP_{delay}$, (**b**) $e_{rise}$. The red * is the discrete value and the left box is the data distribution of $e_{rise}$ under LOS, and the right box is the data distribution of $e_{rise}$ under NLOS.

This paper used $L_{N}$ (the blue horizontal line in Figure 1) to rejudge the true FP ($TFP_{R}$). The positive delay between the $TFP_{R}$ and MFP, which is defined as $TFP_{delay}$, can be calculated as follows:

(3) $$TFP_{delay}=MFP-TFP_{R}=MFP-argmin\left(r\left(t\right)\geq L_{N}\right)$$

where *t* is the time of the CIR value that exceeds the threshold $L_{N}$. In theory, the CIR in the LOS ENS is stable, with only natural fluctuations and no UWB signal. Hence, both *S* and $CDF_{ENS}^{-1}\left(0.9\right)$ can accurately evaluate the signal noise level. Since the CIR rises sharply and approaches a vertical line under LOS conditions, $TFP_{delay}$ is very small at 0.1289, as shown in Figure 1a. In NLOS, the fixed NTM value is too large and can cause *L* to increase, leading to a wrongly classified weakened TFP and high-energy MPs as the ENS, resulting in a vicious circle of increasing *S* and *L*. At this point, there is a large difference between the MFP identified by the LDE and the TFP. In this paper, we use $CDF_{ENS}^{-1}\left(0.9\right)$ of $L_{N}$ to evaluate the real signal noise level and avoid the high-energy TFP and MP from UWB, along with a multiplier of 3 to avoid signal noise mutation. Moreover, the weakened TFP slowly increases the CIR, leading to a further enlarging of $TFP_{delay}$ under NLOS conditions with a value of 4.4059, as shown in Figure 1b. Figure 2a shows the distribution of $TFP_{delay}$ in different channel environments. The total data of the CDF is 33,000, derived from the actual measurements of four static experiments consisting of two experimental scenarios and three obstacles. Overall, $TFP_{delay}$ in NLOS is higher than in LOS, with a maximum value of 63.24 in the multiscene dataset, which is much higher than the 16.22 in LOS. In LOS, 95.8% of $TFP_{delay}$ is concentrated at 0.5 and below, whereas this number decreases to 75% in NLOS. When the range of $TFP_{delay}$ extends to 1, the proportion of NLOS is only 84%, which is still much lower than the 96% in LOS. Combining the principle analysis and data distribution, the proposed new CIR feature, $TFP_{delay}$, can be effectively used to distinguish the channel state.

(2) Energy Rise ($e_{rise}$)

The typical energy-based CIR features are statistical characteristics of the CIR sequence that correspond to different UWB signal types or according to the correlation between the energy and rapid rise of individual CIR features, such as MFP and SP. However, these parameters do not take into account the channel information contained in the signal noise energy from the environment. To address this, we propose a new parameter called energy rise ($e_{rise}$), which considers the energy difference between the UWB’s FP signal and signal noise level, as follows:

(4) $$e_{rise}=F2-S$$

where F2 is the CIR of the second index after the MFP, as shown in Figure 1, and *S* is the same as in (1). In LOS, the CIR rises rapidly and reaches maximum amplitude within three units after the MFP. Empirically, the maximum amplitude is likely to be F2 in LOS, as shown in Figure 1a, which is highly distinguishable from the signal noise level [48]. However, in NLOS, due to the high delay in the SRS caused by obstacle interference with the UWB signal, the energy cannot reach its maximum value at F2, as shown in Figure 1b. The difference between F2 and *S* under NLOS conditions is much smaller than that under LOS conditions. As shown in Figure 1a, the $e_{rise}$ value in LOS is 14,627, which is 10,964 higher than that in NLOS (3663). Moreover, Figure 2b and Table 1 show that the $e_{rise}$ values in LOS under multiple scenarios (same as $TFP_{delay}$) are significantly higher than those in NLOS. Under LOS, the $e_{rise}$ values are mostly clustered between $12.3\times 10^{4}$ and $13.6\times 10^{4}$, whereas under NLOS, they are clustered between $7.5\times 10^{4}$ and $11.1\times 10^{4}$. The average (median) $e_{rise}$ value in NLOS is 8848 (9856), which is much lower than 12,939 (13,005) in LOS. Data with $e_{rise}$ values below $\times 10^{5}$ mostly correspond to the NLOS channel.

### 3.2. Fuzzy Credibility Evaluation

**Figure 3:**
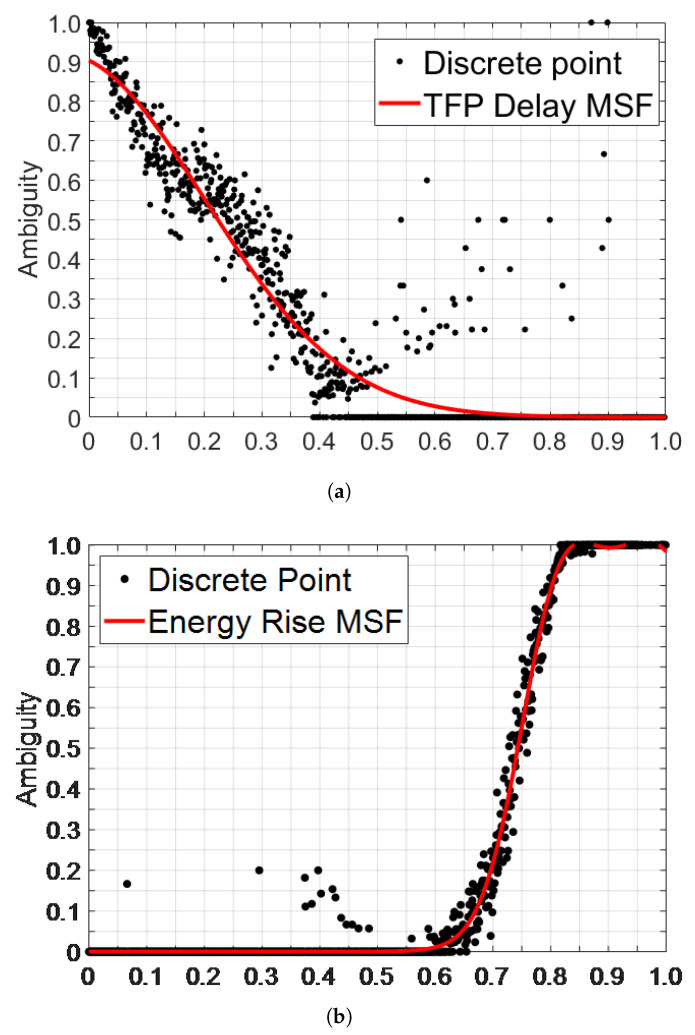
The MSFs of the new CIR features: (**a**) $TFP_{delay}$, (**b**) $e_{rise}$.

In different scenarios, the same CIR feature value may indicate an opposite channel state under different external conditions. This is mainly because obstacles with different materials and thicknesses attenuate signals in different ways, causing fluctuations in the numerical values of CIR features. This phenomenon is one of the reasons why the classification accuracy of CIR features fluctuates with changes in external conditions. To improve the environmental robustness of CIR features and identification algorithms, this paper introduces the concept of fuzziness and employs fuzzy theory to extract channel information from the parameters. Rather than ultimately attributing the CIR feature with a specific value ($S=s_{1},s_{2},\cdots ,s_{I}$) to LOS or NLOS, this paper assigns a new result ($f_{los}\left(s_{i}\right),i=1,2,\cdots ,I$) to measure the probability of the data’s channel environment being identified as LOS. $f_{los}\left(s_{i}\right)$ is determined by the membership function (MSF), which is defined using statistical information or experience and consists of three steps. First, the CIR feature values are compressed to [0,1] to obtain the normalized $s_{i}^{\prime }$ from $s_{i}$ by leveraging large amounts of off-line data. This can reduce the influence of varying parameter dimensions on the defuzzification operation. Second, the statistical probability method is used to determine the ambiguity of $s_{i}^{\prime }$ by counting the number of occurrences of the same value in the LOS (${N_{s_{i}^{\prime }}}^{los}$) and NLOS (${N_{s_{i}^{\prime }}}^{nlos}$) channels. The ambiguity of $s_{i}^{\prime }$ is ${N_{s_{i}^{\prime }}}^{los}⁄\left({N_{s_{i}^{\prime }}}^{los}+{N_{s_{i}^{\prime }}}^{nlos}\right)$. Third, as more data are collected and parameter intervals are refined, a larger number of discrete points can be obtained, as illustrated in Figure 3. This paper employs Gaussian fitting to determine the MSF of new CIR features, as depicted by the red curve in Figure 3. More information on the MSF and other parameters can be found in [49,50].

The above operations are fuzzy operations for a single CIR feature. The operation to obtain the fuzziness of the feature vector set is as follows, which is called defuzzification:

(5) $$F=CP^{\prime }\circ R$$

where *F* and $CP^{\prime }$ are two fuzzy sets of the credibility evaluation domain and normalized parameter domain, respectively; *R* represents the above fuzzy sets’ relationship; and ‘∘’ is the fuzzy operator used to obtain the evaluation results according to the information of the fuzzy sets. Suppose there are *m* kinds of data, and each combination $g_{m}$ has *J*$CP^{\prime }$, as follows:

(6) $$CP_{m}^{\prime }=cp_{m,j}^{\prime },j=1,2,\cdots ,J$$

where $cp_{j}^{\prime }$ is the normalized value of $cp_{j}$. The correlation matrix *R* is defined as $R_{m}$ = ${\left\{r_{m,j}\right\}}_{m=1,j=1}^{\left(M,J\right)}$, and the credibility evaluation of the CIR feature vector is evaluated using $F_{m}$, as follows:

(7) $$F_{m}=CP_{m}^{\prime }\circ R_{m}=\left[cp_{m,1}^{\prime },cp_{m,2}^{\prime },\cdots ,cp_{m,J}^{\prime }\right]\circ \left[r_{m,1},r_{m,2},\cdots ,r_{m,J}\right]$$

where $r_{m,j}$ is calculated differently according to different fuzzy operators. This paper uses the average ($F^{a}$) and weighted-average ($F^{w-a}$) values of CIR features to evaluate the ambiguity of the vector, as follows:

(8) $$F_{m}^{a}=CP_{m}^{\prime }\circ R_{m}=\sum_{j=1}^{J}cp_{m,j}^{\prime }*r_{m,j}=\sum_{j=1}^{J}cp_{m,j}^{\prime }*f_{los}\left(cp_{m,j}^{\prime }\right)$$

(9) $$F_{m}^{w-a}=CP_{m}^{\prime }\circ R_{m}=\sum_{j=1}^{J}cp_{m,j}^{\prime }*r_{m,j}=\sum_{j=1}^{J}cp_{m,j}^{\prime }*f_{los}\left(cp_{m,j}^{\prime }\right)/\sum_{j=1}^{J}f_{los}\left(cp_{m,j}^{\prime }\right)$$

## 4. Proposed Method

**Figure 4:**
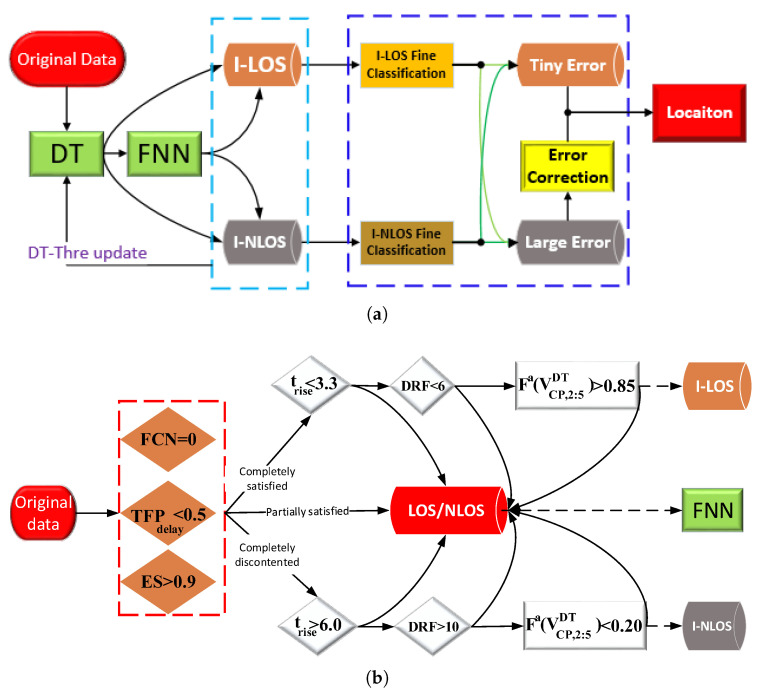
The complete process of UWB channel identification and error correction: (**a**) the two-step channel identification algorithm for UWB data via the DT and FNN, as well as the error correction process based on the channel identification results; (**b**) the DT process in Figure (**a**), including the CIR features used and the thresholds.

To precisely determine the coordinates of unknown locations, the accurate identification of channel states is essential for achieving exceptionally high-precision LOS ranging results. High-precision channel identification can leverage the advantages of UWB and ensure effective ranging error mitigation. In addition, this paper refines the classification of LOS and NLOS ranging errors and develops corresponding error mitigation algorithms and recall mechanisms. The overall architecture is illustrated in Figure 4a.

In the process of NLOS identification, this study employs the DT and FNN to ascertain the channel state. Initially, data exhibiting characteristic LOS and NLOS CIR waveform features across all three communication stages are pre-extracted. Additionally, the fuzzy credibility evaluation of the vector $V_{CP}$ is integrated into the DT to augment identification accuracy. As illustrated in Figure 4b, the data are segmented into three categories: identified LOS (I-LOS), identified NLOS (I-NLOS), and ambiguous LOS/NLOS. Both the I-LOS and I-NLOS datasets undergo direct optimization through the ranging error mitigation algorithm, bypassing the need for granular identification via the FNN. For ambiguous LOS/NLOS data, an FNN equipped with self-learning capabilities is utilized to finalize the identification outcome. In the realm of ranging error mitigation, this paper introduces novel definitions for LOS and NLOS errors, grounded in channel characteristics and innovative CIR features. Correction strategies are refined for various I-NLOS error datasets, and a ranging recall mechanism is established. Furthermore, by classifying ranging outcomes, this study precisely corrects misclassified NLOS errors and certain genuine LOS errors within the I-LOS dataset. This approach minimizes the impact of channel identification accuracy on location performance and guarantees the precision of data directly employed for positioning.

### 4.1. Channel State Identification Algorithm

(1) Step 1: Decision tree for pre-extraction

In the LOS environment, there are no obstacles between devices, and the receiver can accurately identify UWB signals with the shortest path and highest link quality. By analyzing the typical difference features in three stages in LOS and NLOS environments, we select the false crest number (FCN), $TFP_{delay}$, CIR rise time ($t_{rise}=SP-MFP$), energy saturation (ES), and the difference between the receive and FP power levels (DRF) to form the vector ($V_{CP}^{DT}$) of the DT. As shown in Figure 1, we record the number of CIRs in the ENS that exceed the FCN threshold (black horizontal line) as the FCN [51], which may include misjudged UWB signals. The threshold (0.6∗L) [52] can effectively distinguish between UWB signals and abrupt signal noise. In most environments, the FCN is lower (FCN=0 in Figure 1a) and much smaller than that in NLOS environments (FCN=3 in Figure 1b).

DTs [7] offer interpretability, handle non-linearity, require no feature scaling, accommodate both numerical and categorical data, and provide feature importance, making them advantageous for classification tasks. As shown in Figure 4b, in this paper, combining the DT with the time domain, energy, and proposed new CIR features extracted from the key UWB communication stages (ENS, SRS) can quickly and accurately identify and pre-extract data with typical LOS or NLOS channel characteristics. Given that CIR fluctuations are theoretically stable without significant energy anomalies in the LOS ENS, the initial state of FCN is set to 0. Taking into account the system error of the LDE [46] and the acceptable ranging error of the trilateral positioning algorithm [19], the initial threshold for $TFP_{delay}$ is established at 0.5. Based on experience and existing references [48,52], instances where $t_{rise}<3.3$ are classified as LOS, and those where $t_{rise}>3.3$ are classified as NLOS. Additionally, DRF<6 indicates LOS, whereas DRF>10 suggests NLOS. Moreover, the LOS/NLOS boundary for ES is 0.9. To improve the reliability of the pre-extracted data, we introduce the ambiguity of $F^{a}\left(V_{CP,2:5}^{DT}\right)$ to ensure accuracy. Following pre-extraction, the FNN algorithm processes the data labeled as LOS/NLOS to ascertain the channel state. As illustrated in Figure 4, to enhance the algorithm’s robustness to external conditions, every 30 data points with the final determined channel states are added to the respective threshold datasets. The thresholds are then updated by calculating the average. Data that do not meet the initial thresholds are removed from the dataset and not used for updating.

(2) Step 2: FNN for identification of remaining data

FNNs [53] model, analyze, and solve nonlinear problems by simulating the learning process of the human brain using interconnected artificial neurons. In this paper, we construct a three-layer, fully connected feedforward neural network with ten neurons in each layer. The input parameter feature set is $V_{CP}^{FNN}$ = [FCN; $TFP_{delay}$; $e_{rise}$; $t_{rise}$; ES; root-mean-square delay ($\tau_{rms}$); kurtosis (*k*) [28]; mean excess delay ($\tau_{med}$); DRP; the standard deviation of CIRs ($\sigma_{r}$)]. Unlike the traditional FNN, we initialize the model with the ambiguity of the training dataset as the initial weight, as follows:

(10) $$y_{n}=F\left(\sum_{k=1}^{K}\omega_{k}*x_{k}+b\right)$$

where $x_{k}$ is the $k^{th}$ input of the $n^{th}$ neuron; *K* is the number of inputs; $y_{n}$ is the corresponding output of vector $X=[x_{1},x_{2},\cdots ,x_{K}]$; *b* is the bias value; $\omega_{k}$ is the input weight, which controls the influence of the last neuron on the output function; and F is the activation function used in the neuron. The activation function computes the output from the weighted sum of inputs and can be a sigmoid function, hyperbolic tangent function, softmax function, or rectifier function. The supervised FNN improves its performance by ‘learning’ from a known dataset. Each element is defined as the derivative of the error measure with respect to a parameter by backpropagating the gradient vector to update the weights of the synaptic network. The error cost function is expressed in this paper as the difference between the target output (*t*) and *y*, using the cross-entropy loss, as follows:

(11) $$Cost=-\frac{1}{N}\sum_{n=1}^{N}\left(y_{n}logt_{n}+\left(1-y_{n}\right)log\left(1-t_{n}\right)\right)$$

where n=1,2,⋯,N is the number of output neurons, and Cost is the cost of the training dataset. The purpose of ‘learning’ is to update parameters, such as the weight and deviation from the minimum (local minimum) parameters. During neural network training, we use the gradient descent (GD) method to iteratively update the initial weight, which is defined as follows:

(12) $$\omega^{\left(l+1\right)}=\omega^{l}-\eta \nabla Cost\left(\omega^{\left(l\right)}\right)$$

where η is the learning rate, that is, the speed at which the neural network approaches the local minimum of the cost function. The initialization of model weights is crucial for training the network, as improper initialization can lead to a large variance of hidden layer data, causing gradients to vanish prematurely when passing through the nonlinear sigmoid layer (where the derivative is close to 0). Standard initial weight methods include zeros, ones, Xavier, He, narrow-normal, and orthogonal [54,55]. To enhance the relevance between the initial thresholds and the classification purpose and to reduce dependence on the distribution of the training set data, this paper proposes using the centroid fuzziness of a single CIR feature in the training set as the initial weight, defined as follows:

(13) $$\omega_{k}^{\left(0\right)}={\left[\begin{array}{c} \;F^{\left(w-a\right)}\left(V_{CP,1}^{FNN}\left(1:P\right)\right)\\ F^{\left(w-a\right)}\left(V_{CP,2}^{FNN}\left(1:P\right)\right)\\ \cdots \\ F^{\left(w-a\right)}\left(V_{CP,10}^{FNN}\left(1:P\right)\right) \end{array}\right]}^{T}$$

where *P* is the number of off-line datasets, and $V_{CP,i}^{FNN}\left(1:P\right)$ is all data in the off-line dataset for the $i^{th}$ CP in $V_{CP,i}^{FNN}$. This paper uses the stochastic gradient descent (SGD) method by randomly selecting samples to update parameters and iterate over others repeatedly. It can accelerate the convergence speed, reduce the computational power consumption of similar samples, and minimize memory consumption. Each weight is updated based on the partial derivative of the cost function, as follows:

(14) $$\omega_{k,n}^{\left(l+1\right)}=\omega_{k,n}^{\left(l\right)}-\eta \left(\partial Cost_{p}/\partial \omega_{k,n}^{\left(l\right)}\right)$$

where $\omega_{k,n}$ is the weight of the $n^{th}$ neuron for the $k^{th}$ input. Based on the chain rule, we can calculate the weight update rules for all layers of the FNN as follows:

(15) $$\omega_{k,n}^{l+1}=\omega_{k,n}^{l}-\eta \left[E_{n}y_{n}\left(1-y_{n}\right)x_{k}\right]$$

where $E_{n}=\partial Cost_{p}/\partial y_{n}=y_{n}-t_{n}$ is the error term of the $n^{th}$ neuron in the output layer.

### 4.2. Classification and Correction of Ranging Errors

Figure 4a illustrates the application of the two-step NLOS channel identification algorithm introduced in this study, which categorizes UWB data into two groups: I-LOS and I-NLOS. The key difference between these categories and traditional LOS and NLOS data is that the channel identification algorithm does not fully and accurately discern the UWB channel environment. This leads to a certain degree of misclassification in both I-LOS and I-NLOS data. Traditional error correction algorithms exclusively utilize I-LOS data for positioning, but this approach is flawed due to the inclusion of incorrectly identified NLOS data, which compromises positioning accuracy. Moreover, the I-LOS dataset also encompasses some data with substantial errors. Regarding I-NLOS data, traditional algorithms attempt complete correction, which presents several issues. Firstly, the inclusion of some LOS data in I-NLOS means that their full correction can inadvertently reduce ranging accuracy. Secondly, given the inherent capabilities of UWB signals, certain NLOS conditions still yield ranging results that satisfy accuracy requirements for positioning; correcting these data can result in a loss of both accuracy and the inherent benefits of UWB signals. Thirdly, existing error correction algorithms fail to integrate with UWB waveforms, relying instead on a uniform model for ranging correction, which significantly diminishes the effectiveness of error correction. In response to these challenges, this paper proposes a novel approach involving the classification of ranging errors and a model for the recall and correction of ranging errors, which also incorporates I-LOS ranging results.

**Figure 5:**
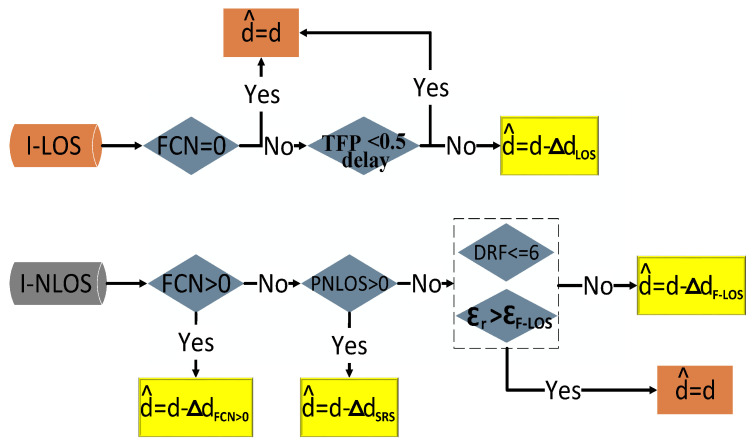
The overall framework for UWB ranging error correction, including the recall of I-LOS and I-NLOS ranging results, the mitigation process, and the corresponding CIR features and thresholds.

As shown in Figure 5, based on the characteristics of theoretically stable LOS ENS signals with almost no high-energy CIR, this paper classifies I-LOS data using FCN and the new proposed CIR feature ($TFP_{delay}$) as the threshold criteria. When I-LOS data satisfy the threshold parameters, as depicted in Figure 5, the relevant results are retained for direct use in positioning. Data not meeting these criteria are defined as $\Delta d_{LOS}$ and require correction prior to their use in positioning. For I-NLOS data, this paper selects four feature parameters—FCN, PNlos, DRF, and $\varepsilon_{r}$—and employs a step-by-step classification approach to approximate theoretical LOS data. Initially, if the FCN value of I-NLOS data exceeds 0, it suggests the presence of high-energy CIRs, such as UWB signals, in the ENS. This data subset, labeled as $\Delta d_{FCN>0}$, necessitates correction, whereas the remaining data align with the characteristics of the LOS ENS. The subsequent parameter, PNlos, relates to the UWB’s SRS. Under LOS conditions, a marked increase in the SRS results in PNlos being 0, and NLOS otherwise. Data falling into this category, termed $\Delta d_{SRS}$, also require correction, whereas the rest conform to the characteristics of the LOS SRS. Finally, the energy features, DRF and $\varepsilon_{r}$, are utilized for the ultimate differentiation. If the energy features of I-NLOS data align with the criteria shown in Figure 5, the ENS, SRS, and energy aspects of such data collectively meet LOS characteristics, warranting their recall and use in positioning without further correction. Data not meeting these criteria are classified as $\Delta d_{F-LOS}$ and necessitate correction before their use in positioning applications. In the process of ranging error mitigation, we employ LS-SVM [21,29], a supervised learning technique used for classification and regression, with the estimated TFP as a core parameter. The following are mitigation algorithms for different types of ranging errors:

(1) I-LOS Ranging Errors

To improve the accuracy and stability of positioning, it is essential to identify and correct ranging errors in I-LOS data, which include misjudged NLOS ranging and partial LOS data with high ranging errors due to environmental factors or the movement of people. I-LOS data with FCN=0 indicate no energy outlier in the ENS, and the UWB TFP signal is identified accurately, so no mitigation is required. Data with FCN>0 and $TFP_{delay}\leq 0.5$ indicate signal noise mutation, and the TFP is still higher than the dynamic threshold *L*. Therefore, they do not need to be mitigated. For the remaining data with FCN>0 and $TFP_{delay}>0.5$, this paper mitigates the ranging error due to $TFP_{delay}$ and the first $FCN(FCN_{1})$. Δd represents the amount of error correction, and the lower-right corner indicates the type of error that it belongs to. For instance, $\Delta d_{LOS}$ denotes the ranging distance correction for I-LOS ranging errors, and its model is as follows:

(16) $$\Delta d_{LOS}=LF\left(TFP_{delay},FCN_{1}\right)*c*time$$

where time=1/499.20/1000000/128 is the UWB hardware time and real-time conversion parameters, and LF denotes LS-SVM, which is used to fit the planned inputs and outputs.

(2) I-NLOS Ranging Errors

The typical LOS CIR waveform is characterized by stable ENS fluctuations and fast-rising SRS values, which correspond to the FCN and the probability of NLOS (PNLOS) parameters [52], respectively. As shown in Figure 5, this paper divides the NLOS ranging errors into three categories, where $\hat{d}$ and *d* represent the mitigated and original ranging distances, respectively.

(i)
$NLOS_{ENS}$

The NLOS ranging error with FCN>0 is identified as $NLOS_{ENS}$. This type of error arises from a weakened UWB signal that fails to meet the dynamic *L*. The LDE algorithm incorrectly identifies a high-energy MP signal as an FP signal. This paper calculates the mean value of the parameter $TFP_{delay}$ and all FCNs ($FCN_{all}$) and combines them with the total CIR energy ($\varepsilon_{r}$) and maximum CIR amplitude ($r_{max}$) to construct the ranging distance correction for $NLOS_{ENS}$, $\Delta d_{FCN>0}$, as follows:

(17) $$\begin{array}{c} \Delta d_{FCN>0}=\mathrm{LF}\left(mean\left(TFP_{delay},FCN_{all}\right)*c*time,\varepsilon_{r},r_{max}\right) \end{array}$$

(ii)
$NLOS_{SRS}$

$NLOS_{SRS}$ indicates that the data do not exhibit typical NLOS characteristics in the ENS (FCN=0) but instead have a high delay in the SRS (PNLOS>0). This scenario is characterized by a severe multipath effect, resulting in the LDE algorithm incorrectly identifying an MP signal as an FP signal. This paper performs error mitigation based on the energy and LDE parameters. The correction mode ($\Delta d_{SRS}$) for $NLOS_{SRS}$ ranging errors is as follows:

(18) $$\Delta d_{SRS}=\mathrm{LF}\left(TFP_{delay}*c*time,\tau_{med},RPL\right)$$

(iii)
$NLOS_{F-LOS}$

These NLOS ranging errors exhibit apparent characteristics of the LOS ENS (FCN=0) and SRS (PNLOS=0), thus classified as $NLOS_{F-LOS}$. When combined with the channel environment identified as NLOS, we can conclude the following two phenomena. First, the TFP is entirely blocked, and the hardware only receives the reflections of the UWB signal over the delay. Second, the LDE can still accurately identify UWB signals, which are misjudged LOS data or due to the weak attenuation of signals occluded by obstacles. The accuracy of these ranging results still meets the requirements of positioning. As shown in Figure 1, the CIR between the 500 and 700 unit-time intervals generally only contains environmental noise and is closer to the TFP, which has a greater impact on the LDE algorithm and results in notable differences between the LOS and NLOS channels. This paper segments the threshold $\varepsilon_{F-LOS}=2\times 10^{5}$, based on $\varepsilon_{r}$ of the CIR in the ENS between 500 and 700, as well as DRF, to distinguish between the above two phenomena. The value of the threshold $\varepsilon_{F-LOS}$ is based on the actual measurements of four static experiments consisting of two experimental scenarios and three obstacles. Data exceeding $\varepsilon_{F-LOS}$ are not within the mitigation range, and the model $\Delta d_{F-LOS}$ mitigates the remaining $NLOS_{F-LOS}$ ranging errors by combining $TFP_{delay}$, $t_{rise}$, and FPPL as follows:

(19) $$\Delta d_{F-LOS}=\mathrm{LF}\left(TFP_{delay}*c*time,t_{rise},FPPL\right)$$

## 5. Experiments

In this paper, we designed four static experiments, denoted as STA, to test the accuracy of the NLOS identification algorithm and the performance of the ranging error mitigation strategy. Additionally, three dynamic experiments, denoted as DYN, were set up to assess the improvements in the final positioning results achieved by the aforementioned algorithm. The hardware employed in these experiments was the DW1000 UWB module, based on IEEE 802.15.4-2011 [56]. The experiments were conducted in two different environments: the UWB test site of the School of Environment and Spatial Informatics, China University of Mining and Technology, denoted as CUMT, and the State Key Laboratory of Satellite Navigation Systems and Equipment Technology, 54th Research Institute of China Electronics Technology Group Corporation, denoted as LAB.

**Figure 6:**
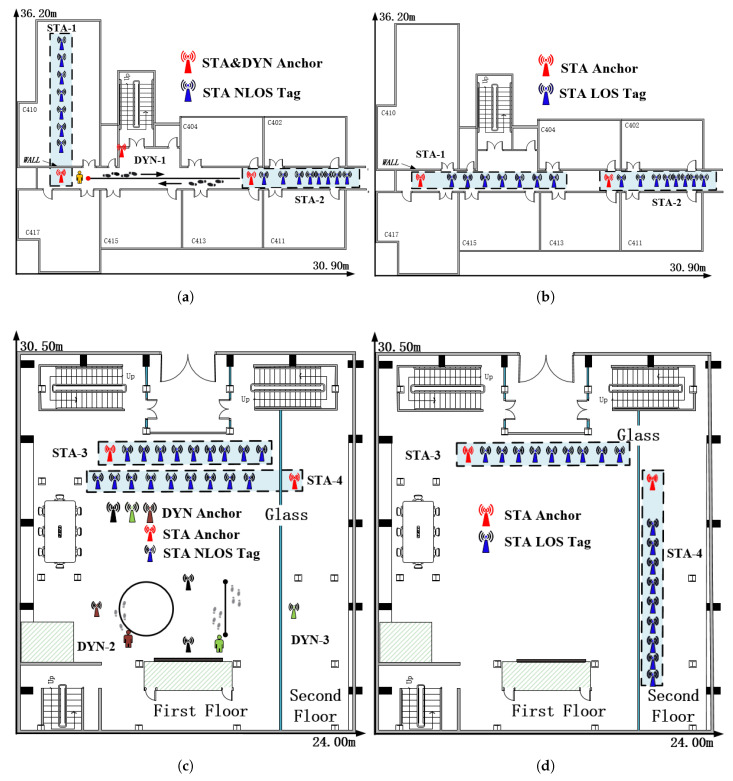
The layout of the anchors at the different test sites: (**a**) the anchor layout of NLOS for the STA and DYN experiments at CUMT, (**b**) the anchor layout of LOS for the STA experiments at CUMT, (**c**) the anchor layout of NLOS for the STA and DYN experiments at LAB, (**d**) the anchor layout of LOS for the STA experiments at LAB.

As shown in Figure 6a, there was an obstacle (wall) between the red fixed anchor and the blue mobile tag in STA-1. For the seven groups of static experiments at different distances, as shown in Table 1, we ensured that the anchor’s position remained unchanged and moved the tag to collect NLOS data. To collect LOS data simultaneously in each group for STA-1, we set up another LOS tag in the corridor, as shown in Figure 6b, to ensure that the distance between the LOS tag and the anchor was the same as that between the NLOS tag and the anchor. The hardware collected 50 data points per minute, and each set of experiments lasted ten minutes. In STA-2 and STA-3, the obstacle was a human. Therefore, for each group, we first collected LOS data. The anchor and tag positions are shown in Figure 6b,d, respectively. Then, we arranged for the experimenter to stand between the anchor and the tag to block the LOS signal and collected NLOS data without moving the tag and anchor. The anchor and tag positions are shown in Figure 6a,c. After collecting one set of LOS and NLOS data, we retained the position of the red anchor, moved the blue tag to the next position, and repeated the above collection steps. In STA-4, the red fixed anchor was on the second floor, and a glass obstacle always existed. The collection strategy was the same as that in STA-1. The UWB’s positions in the LOS and NLOS scenarios are shown in Figure 6c,d, respectively. To ensure consistency with the comparison algorithm [29,30], the training and testing data were split into a 70:30 ratio from the acquired data. This split was utilized to obtain the existing and proposed CIR features ($TFP_{delay}$, $e_{rise}$) and to train the NLOS identification and ranging error mitigation model. The experimental details are shown in Table 2.

### 5.1. LOS/NLOS Identification Performance

This paper used TL/TN to represent the number of data correctly identified as LOS/NLOS and FL/FN to represent the corresponding misjudged data. The accuracy and recall rates were used as the evaluation metrics for the algorithm and are defined as follows:

(20) Accuracy=(TL+TN)/(TL+FL+TN+FN)

(21) $$Recall_{LOS}=TL/\left(TL+FL\right)$$

(22) $$Recall_{NLOS}=TN/\left(TN+FN\right)$$

(i) The New CIR Features and Optimization of the Existing CIR Features

Based on the previous analysis, the number of CIRs used to calculate the existing CIR features and their communication stages can affect their performance. This paper used LS-SVM to evaluate the performance of each CIR feature. In the ENS, the number of CIRs used to calculate each CIR feature started from the MFP and gradually increased to 500 indexes. In the SDS, the number of CIRs used to calculate the CIR features started from the SP and gradually increased to 500 indexes. We optimized the existing CIR features (the receive energy ($\varepsilon_{r}$); the sum of the CIRs ($sum_{r}$); the standard deviation of the CIRs ($\sigma_{r}$); $\tau_{med}$; $\tau_{rms}$; the mean of the CIRs ($mean_{r}$); *k*; and skewness (ske)), as shown in Table 3.

**Figure 7:**
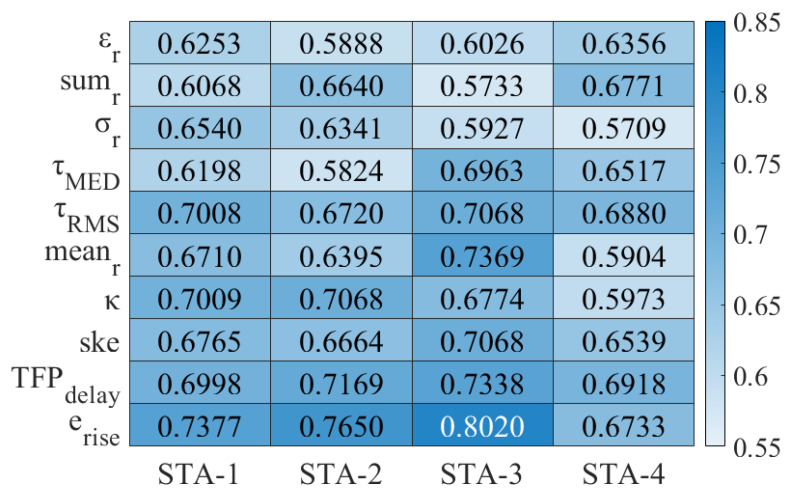
The accuracy of channel identification using the new CIR features ($TFP_{delay}$, $e_{rise}$) and the optimized existing CIR features.

As shown in Figure 7, the newly proposed features, $TFP_{delay}$ and $e_{rise}$, achieved average accuracies of 71.06% and 74.45%, respectively, across different scenarios, with the lowest accuracy recorded as 69.18% for $TFP_{delay}$ in STA-4, and the highest accuracy recorded as 80.20% for $e_{rise}$ in STA-3. The accuracies of the newly proposed features surpassed those of most optimized existing features in each scenario. The feature $TFP_{delay}$ was optimal in STA-2 and STA-4, but its accuracy was close to that of $\tau_{rms}$ and *k* in STA-1 and close to that of $mean_{r}$ in STA-3, all of which were superior to the remaining existing features. However, the accuracies of $\tau_{rms}$, *k*, and $mean_{r}$ were significantly lower than that of the new feature $TFP_{delay}$ in both STA-2 and STA-4, with a maximum difference of 10.14%. Although the accuracy of feature $e_{rise}$ was lower in STA-4, it was the best across the remaining scenarios, leading by up to 9.52% compared to the highest value among the remaining feature parameters. Compared to the existing features, the accuracies achieved by the newly proposed features in this paper were mainly around 70%, whereas some existing features achieved accuracies below 60% and exhibited poorer environmental robustness. The novel feature $TFP_{delay}$ assessed the channel status by leveraging the LDE algorithm’s output, enabling more precise acquisition and filtration of abnormal CIR amid environmental noise. In contrast, the existing timing characteristics ($\tau_{med}$, $\tau_{rms}$, *k*, and ske) merely represented the mathematical statistics of a subset of the CIR sequence, lacking integration with the LDE algorithm and environmental noise considerations. The novel feature $e_{rise}$ enhanced the distinction between UWB signals and environmental noise at the energy level. This was in contrast with the other energy characteristics ($\varepsilon_{r}$, $sum_{r}$, $\sigma_{r}$, and $mean_{r}$), which remained merely statistical analyses of a portion of the CIR, without any association with environmental conditions.

(ii) Two-Step Channel Identification Algorithm

This study validated the efficacy of the two-step NLOS identification algorithm across four STAs with three obstacles, with the findings detailed in Table 4. The algorithm attained an average accuracy of 95.05%, peaking at 98.17%, and consistently maintained accuracies above 93% in challenging environments, such as the narrow corridor in STA-2, which was characterized by severe multipath conditions. The $Recall_{LOS}$ rate averaged 95.72%, marginally surpassing the $Recall_{NLOS}$ rate of 94.15%. This difference is crucial for attaining high-precision ranging outcomes and improving positioning accuracy. Notably, the algorithm exhibited stability and delivered satisfactory performance in STA-4, despite the presence of low signal attenuation coefficients and glass obstacles. Across the four scenarios, compared to LS-SVM and K-NN, which achieved average accuracies of 84.85% and 77.20%, respectively, the proposed two-step algorithm showed significant improvements of 10.20% and 16.25%. Transitioning between scenes diminished the efficacy of the LS-SVM feature vector set due to variations in the layout and obstacle types across the different environments. However, the proposed algorithm enhanced environmental resilience through the integration of scene fuzziness and dynamic update mechanisms, facilitating swift adaptation to new settings. In contrast, K-NN required the continuous addition of reference points to maintain precision, yet the variability of characteristic fingerprint values over time compromised its effectiveness, lacking an adaptive update mechanism. The two-step algorithm’s lowest accuracy of 93.32% was still higher than the CNN’s average accuracy of 92.13%. Conversely, the CNN predominantly focused on estimating the CIR sequence’s timing information, neglecting the impact of energy attenuation from UWB signal obstruction by obstacles. The proposed algorithm adeptly estimated both timing and energy attributes, optimizing the utilization of information across diverse channel conditions. It is noteworthy that the two-step algorithm showed a maximum improvement of 12.67% over LS-SVM in STA-3, and for K-NN, the maximum improvement was 16.21% in STA-1. For $Recall_{LOS}$, which attained high-precision ranging results in STA-1 and STA-2, the CNN and LS-SVM algorithms exhibited similar performance to the two-step algorithm, but in STA-3 and STA-4, the average $Recall_{LOS}$ was lower by 4.13%, with a maximum of 5.60%. The $Recall_{LOS}$ for K-NN was significantly lower, leading to a noticeable loss in high-precision LOS ranging. Regarding the specific NLOS recall metric ($Recall_{NLOS}$), the algorithm presented in this study consistently achieved stable performance, maintaining a rate of 93% and above. In contrast, the performance of the existing algorithms generally fell below 90%, with the lowest recorded at 79.89% for K-NN in STA-4. This represents a significant 13.23% deficit compared to our algorithm. Additionally, the CNN attained a $Recall_{NLOS}$ of 92.62% in STA-3, yet it still trailed our algorithm by 4.72%. Consequently, our algorithm demonstrated superior precision in NLOS identification (recall rate for LOS) compared to the other algorithms. In summary, the proposed algorithm demonstrates superior performance compared to the CNN, LS-SVM, and K-NN algorithms.

**Figure 8:**
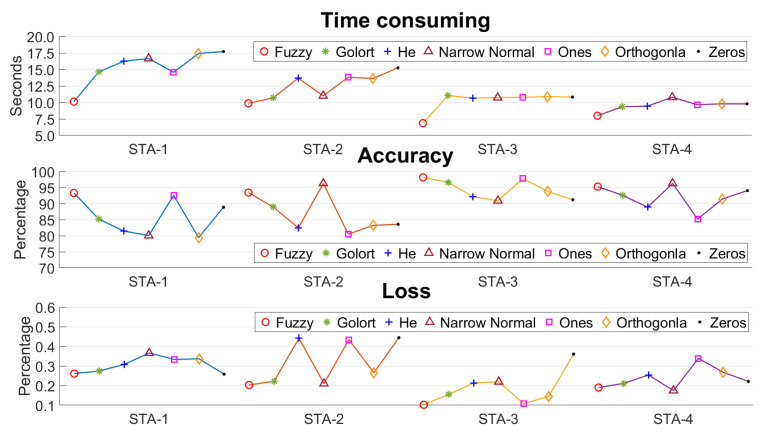
The performance of the FNN using different initial weight methods.

Figure 8 illustrates a comparison between the proposed initial weights and existing methods. Under the same external conditions, $F^{w-a}$ converged to the optimal solution faster and reduced time consumption by 22.15% compared to the average performance of the other algorithms. In terms of accuracy, $F^{w-a}$ exhibited higher precision in most scenarios. Compared to the narrow-normal method, although the precision of $F^{w-a}$ decreased by 1.44% in STA-2 and STA-4, it increased by 10.25% in the other scenarios. Compared to existing methods, our algorithm reduced data loss by an average of 8.5%, with a maximum reduction of 25.82%. In summary, the proposed algorithm achieves lower time consumption and higher accuracy, maximizes data utilization, and exhibits better robustness compared to existing methods.

### 5.2. Ranging Error Mitigation Evaluation

This paper classified ranging errors based on the waveform and selected corresponding CIR feature vectors for different error types. In addition to traditional I-NLOS data, this paper also included part of the I-LOS data in the mitigation scope to improve performance. We used the mean absolute error (mean), standard deviation (STD), and root-mean-square error (RMSE) to measure performance improvements.

**Figure 9:**
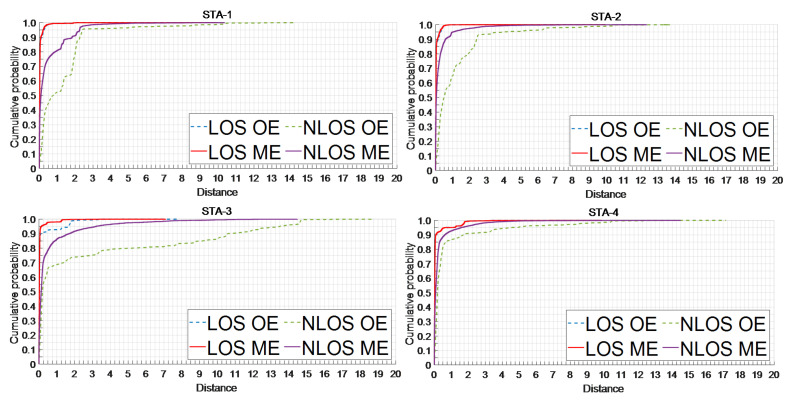
The cumulative distribution functions of the original and mitigated ranging errors across four scenarios.

Table 5 shows that the mean mitigated I-LOS error was 0.065, representing an average reduction of 25.19%. Both the STD and RMSE experienced decreases of 18.02% and 29.23%, respectively. Even in the STA-3 and 4 scenarios with higher errors, the mitigated I-LOS average ranging error remained less than 0.10 m. The CDF depicted in Figure 9 indicates an improved distribution of mitigated ranging errors for I-LOS compared to its original state. The percentage of errors within the centimeter range, denoted as Cm-error, for mitigated ranging averaged 91.37%, which is an increase of 1.89% from the original value, reaching as high as 94.4%. By aggregating the data across the various scenarios, the mean ranging errors for the two types of I-LOS data that did not necessitate mitigation were 0.051 m and 0.056 m, respectively. Ranging errors of around 0.05 m are deemed sufficient for positioning purposes, thereby validating the I-LOS ranging error classification algorithm proposed in this study. The mean error for the remaining ranging errors ($\Delta d_{LOS}$) saw a significant decrease from 1.377 m to 0.683 m, amounting to a 50.4% reduction. Additionally, the algorithm contributed to reductions in the STD and RMSE of 0.165 m and 0.464 m, respectively. It can be seen that the algorithm in this paper has a very significant correction effect on misjudged NLOS data and LOS data with poor original accuracy in I-LOS.

Table 5 demonstrates that the mean mitigated I-NLOS error was 0.407 m, with an STD and RMSE of 0.936 m and 0.968 m, respectively, all lower than 1 m. Notably, even in the challenging environment of STA-3, the mean ranging error post-mitigation was under 0.60 m, with reductions in the STD and RMSE of 67.62% and 64.96%, respectively. The distribution of mitigated I-NLOS errors, as depicted in Figure 9, shows improvement over the original distribution. In the majority of scenarios, it was possible to maintain 90% of the mitigated ranging errors within 1 m. The average percentage of errors within Cm-error was 53.88%, marking an increase of 1.65 times. The ranging error mitigation algorithm ensured that the mitigated ranging accuracy of most NLOS errors was better than 1 m and greatly increased the proportion of high-precision ranging results, which is very beneficial for subsequent positioning. Even in STA-4, characterized by glass obstacles and weak interference signals, the algorithm presented in this study successfully reduced 90% of the ranging errors to 0.70 m. This represents a decrease from the initial average error of 0.7854 m and elevates the proportion of high-precision centimeter-level ranging outcomes to 55.91%.

**Figure 10:**
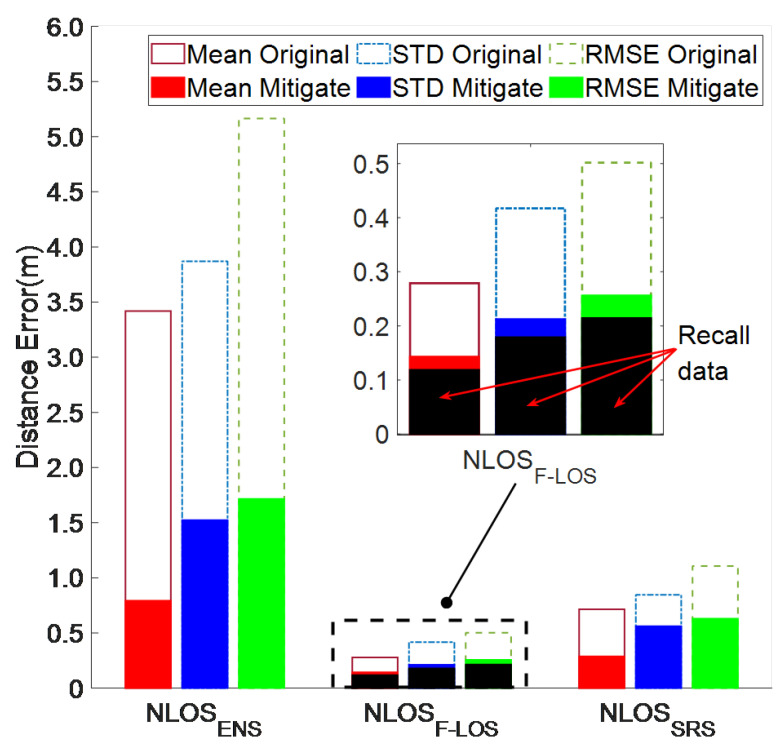
The correction effect of different types of NLOS errors.

Figure 10 shows the performance indicators of different types of NLOS ranging errors across multiple scenarios. The initial mean ranging errors for $NLOS_{ENS}$, $NLOS_{SRS}$, and $NLOS_{F-LOS}$ were 3.419 m, 0.716 m, and 0.278 m, respectively, ranked according to their LOS characteristics from least to most. After mitigation, the mean error for $NLOS_{ENS}$ data was reduced to 0.792 m (76.84%). The STD and RMSE of $NLOS_{ENS}$ decreased by 60.66% and 66.77%, respectively. For the $NLOS_{SRS}$ data, the mitigated mean, STD, and RMSE were better than 1 m, at 0.287 m (59.83%), 0.561 m (33.69%), and 0.630 m (43.12%), respectively. For the fully mitigated NLOS ranging results, the algorithm in this paper significantly reduced the ranging error, ensuring it was less than 1 m. Regarding $NLOS_{F-LOS}$, characterized by the highest similarity to the LOS channel, the mean ranging error of the recall data directly utilized for positioning was 0.118 m, satisfying the requirements for positioning. The mean, STD, and RMSE of the remaining data exhibited improvements of 48.73%, 49.13%, and 49.00%, respectively, after mitigation. Overall, the algorithm presented in this study effectively differentiates various types of NLOS ranging errors, precisely identifies high-precision ranging outcomes, and efficiently corrects the remaining ranging errors.

**Figure 11:**
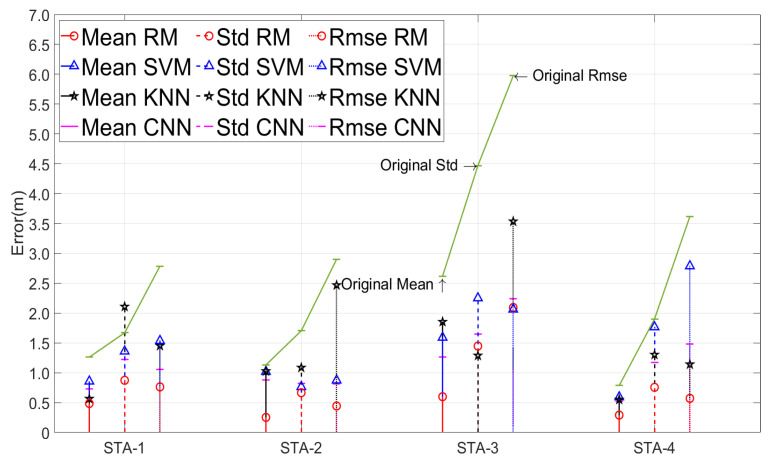
The mitigation performance of the proposed ranging mitigation strategy, denoted as RM, and the K-NN, LS-SVM, and CNN algorithms.

Figure 11 demonstrates that the performance and stability of the proposed ranging mitigation (RM) algorithm surpassed those of the CNN, LS-SVM, and K-NN algorithms. The three algorithms reduced the mean ranging errors by 0.599 m, 0.435 m, and 0.450 m, respectively, which is lower than the 1.043 m in this paper. Only in STA-1 was the correction effect of K-NN close to that of the RM strategy, which was 0.08 m higher. However, the STD of K-NN was 1.23 m and 0.44 m higher than that of ours and the original in STA-1, respectively. The correction of ranging errors in K-NN was predicated upon fingerprint similarity, which linked the ranging outcomes with the outcomes at the reference points. Effective correction occurred only when the ranging device was proximate to a reference point. Furthermore, while enhancing the density of reference points marginally improved K-NN’s performance, it significantly escalated the expenditure of human and material resources. Compared with LS-SVM, our algorithm achieved higher mean, STD, and RMSE values by 43.35%, 26.46%, and 25.83%, respectively. Additionally, the correction effect of LS-SVM was not prominent in STA-4 due to glass obstacles. In comparison to traditional LS-SVM, this study enhanced correction accuracy by refining error categorization and defining specific error correction models. Additionally, a recall mechanism was implemented to retrieve high-precision ranging outcomes, thereby minimizing the risk of inaccurately correcting data and exacerbating errors. On average across the four scenarios, our algorithm’s mean, STD, and RMSE were higher than those of the CNN by 32.51%, 14.06%, and 12.66%, respectively. The overall performance rate of the CNN was higher than that of LS-SVM and K-NN, but it needed to read the full amount of the CIR, which caused a large delay and required more computing power support. The LS-SVM and CNN algorithms are capable of effectively correcting ranging errors; however, they may inadvertently compromise the accuracy of high-precision UWB ranging outcomes that satisfy the criteria for positioning. This issue arises due to their failure to pre-segregate high-precision ranging results, a feature that distinguishes the algorithm discussed in this article. They employ a unified model for error correction, which lacks the necessary precision to identify the TFP signal across various error types, unlike the algorithm presented in this paper that tailors error correction to specific error characteristics. Moreover, the CNN algorithm does not accommodate variations in the actual first-path signal energy. Overall, the error mitigation algorithm presented in this study outperforms existing algorithms in performance, accuracy, and effective utilization of UWB technology’s benefits.

### 5.3. Positioning Experiment

**Figure 12:**
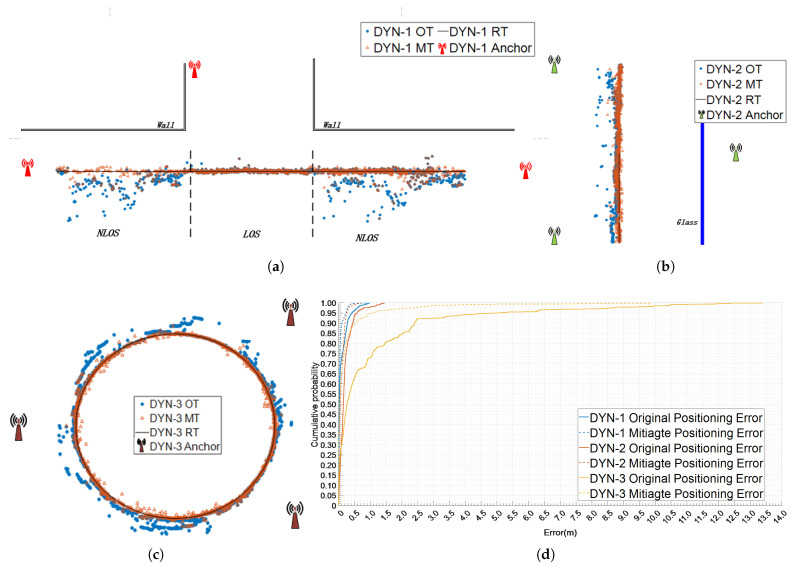
The original trajectory and modified trajectory of the three groups of dynamic positioning experiments. The obstacles are (**a**) walls, (**b**) glass, and (**c**) humans. The position error distributions of the three DYN experiments are shown in (**d**).

This paper conducted three dynamic experiments, denoted as DYN, to assess the efficacy of the proposed NLOS identification algorithm and ranging error mitigation strategy in enhancing positioning accuracy. DYN-1 was executed in a corridor on the fourth floor at the CUMT, featuring a wall obstacle. The participant navigated a round-trip path aligned with the reference trajectory, holding the tag aloft to minimize human-related interference. In Figure 12, the blue points indicate the original trajectory (OT), the orange triangles denote the mitigated trajectory (MT), and the black line represents the reference trajectory (RT). Figure 12a shows the trajectory consisting of two NLOS segments and one LOS segment, with the MT of NLOS more closely aligning with the RT, demonstrating the algorithm’s corrective impact on some LOS points. Despite the high initial positioning accuracy of DYN-1, attributed to a predominance of LOS results, the algorithm introduced in this study further reduced the positioning error from 0.0953 m to 0.0424 m (a 58.58% improvement) and enhanced the centimeter-level positioning accuracy, as shown in the CDF in Figure 12d, by 16.48%. This demonstrates that the algorithm presented in this study effectively differentiated between LOS and NLOS data and further refined the LOS ranging outcomes, thereby significantly enhancing positioning accuracy. DYN-2 involved one anchor positioned on the second floor behind glass in the LAB, with the remaining two anchors on the first floor. The RT was a straight line, traversed by the participant in two round-trip paths. Figure 12b highlights the significant error mitigation, aligning closely with the RT. Figure 12d shows the error reduction from 0.2014 m to 0.0834 m (58.59%) and the rise in the centimeter-level error points from 33.54% to 65.30%. DYN-3, conducted on the LAB’s first floor with a human obstacle, featured a circular RT. The participant’s two round trips are shown in Figure 12c, where the corrected positioning closely follows the RT, albeit with some overcorrection. The CDF in Figure 12d shows an error reduction from 1.0329 m to 0.5237 m (49.30%) and an increase in the meter-level error from 73.03% to 94.83%. Table 6 summarizes the specific ranging errors before and after mitigation, indicating an average decrease in the mean by 54.46%, STD by 54.69%, and RMSE by 52.63%. The positioning results for DYN-2 and DYN-3 do not include scenarios where the ranging results were both LOS. In DYN-3, given that the UWB was positioned at the human-chest level, two out of three ranging results may represent NLOS at certain angles, resulting in a higher proportion of NLOS outcomes compared to DYN-2, and consequently, lower initial positioning accuracy. Nevertheless, in such scenarios, the algorithm presented in this study enhanced meter-level positioning accuracy by over 20%, nearing 95%. The proposed algorithm effectively differentiated between LOS and NLOS ranging results. Through the classification and recall mechanism of ranging errors, it leveraged the capabilities of UWB technology to preserve high-precision ranging outcomes, thereby elevating overall ranging and positioning accuracy. This approach is applicable in both exclusive NLOS and mixed LOS/NLOS environments. The K-NN algorithm enhanced positioning accuracy through the establishment of preset reference points. However, its accuracy fell short of the algorithm presented in this study, attributed to limitations in cost and the quantity of reference points. It merely adjusted the positioning outcome to approximate proximity to the closest reference point, instead of implementing effective corrections. As shown in Table 6, this study’s algorithm surpassed K-NN by 16.32% in RMSE performance, and its advantage in both the mean and STD metrics exceeded 20%. The LS-SVM and CNN algorithms enhanced positioning accuracy by identifying channels and correcting NLOS ranging errors. Regarding the mean accuracy metric, the algorithm discussed in this study outperformed the LS-SVM and CNN algorithms by 17.06% and 13.90%, respectively. For the STD and RMSE metrics, this algorithm surpassed LS-SVM by over 15% and the CNN by more than 8%. However, due to their failure to adjust I-LOS results, their performance in DYN-1 significantly lagged behind the algorithm presented in this study. The LSTM and CNN algorithms effectively mitigated ranging errors and enhanced positioning accuracy. However, unlike the algorithm discussed in this article, they directly utilized I-LOS ranging for positioning. This approach led to significant inaccuracies due to the misclassification of NLOS results and the utilization of low-precision LOS data, thereby making the enhancement of positioning accuracy and error correction heavily reliant on the precision of channel identification. The LSTM–Extended Kalman Filter (EKF) algorithm detected NLOS results using LSTM and employed the EKF to refine the ranging and positioning outcomes. Across three performance metrics, this algorithm exhibited improvements of 8.58%, 6.48%, and 6.39%, with a peak in the average accuracy improvement at 9.04%. The EKF’s smoothing capability enabled the use of LOS results to enhance the NLOS ranging outcomes. Consequently, the enhanced positioning accuracy of LSTM-EKF in DYN-1, characterized by a high prevalence of LOS results, significantly surpassed that of the LS-SVM, CNN, and K-NN algorithms, closely approximating the performance of the algorithm discussed in this study. Nonetheless, as the ratio of LOS diminished, its contribution to positioning accuracy in DYN-2 and DYN-3 declined, and the disparity with the algorithm presented in this paper became more pronounced. The absence of a need for ranging error correction rendered LSTM-EKF more efficient in operation compared to the algorithm discussed in this paper. Yet, its applicability in positioning scenarios was constrained in comparison. For instance, an increase in the NLOS ratio within the test results led to a swift decline in positioning accuracy. Moreover, the outright deletion of NLOS ranging outcomes neglected the unique advantages offered by UWB signals. This observation underscores the high accuracy and environmental adaptability of the algorithm introduced in this study, highlighting its minimal reliance on the proportion of LOS ranging outcomes. In comparison to existing advanced algorithms, the algorithm presented in this study demonstrates superior performance and enhanced environmental robustness.

## 6. Conclusions

This study segments the UWB signal communication process into three stages, optimizes the existing CIR features, and proposes two new CIR features with stronger robustness. By pre-classifying data with typical LOS/NLOS features using a low-computational DT, subsequent algorithms can reduce their computational load while improving accuracy and stability. Furthermore, the integration of fuzzy logic bolsters the DT’s reliability and optimizes the initial weights of the FNN, thereby augmenting the final accuracy. Additionally, the DT threshold is adjusted based on definitive outcomes within specific constraints, improving the efficacy and robustness of the two-step NLOS identification process. This NLOS identification process achieves an accuracy of 95.05%, with a peak at 98.17%. Both $Recall_{LOS}$ and $Recall_{NLOS}$ surpass 92.5%, with an average $Recall_{LOS}$ of 95.71% across various scenarios, aiding in the preservation of high-precision ranging outcomes. In the UWB ranging error mitigation strategy, the system uses CIR features to classify the ranging errors, extract the high-precision ranging results, and formulate error-specific strategies. The mean errors of mitigated ranging for I-NLOS and I-LOS are less than 0.6 m and 0.1 m, respectively, ensuring that more than 90% of the data are better than 1 m. In mitigated I-NLOS, centimeter-level data represent more than 50% of the data, whereas in I-LOS, they exceed 94%. Coupled with the aforementioned algorithm, the system significantly enhances dynamic positioning accuracy across multiple scenarios by an average of 54.47% and reduces the STD and RMSE by more than 52%.

## Figures and Tables

**Table 1:** Comparison of $e_{rise}$ values under LOS and NLOS scenarios.

	Mean	Median	25%	75%
LOS	12,939	13,005	12,308	13,639
NLOS	8848	8848	7544	11,021

**Table 2:** The distances between the anchor and tag in the four STAs.

	Location	Obstacle	Distances
STA-1	CUMT	Wall	3.09, 4.95, 7.03, 9.10, 11.00, 13.01, 15.08
STA-2	CUMT	Human	1.01, 3.21, 5.10, 6.08, 7.15, 8.14, 9.18, 10.22
STA-3	LAB	Human	1.10, 2.29, 3.56, 4.80, 6.01, 7.14, 8.50, 9.56, 10.68
STA-4	LAB	Glass	1.60, 3.04, 4.10, 5.21, 6.38, 7.56, 8.64, 9.49, 10.86

**Table 3:** Optimization of the numbers and sources of CIRs utilized for existing parameter calculations.

CP	Stage	Number	CP	Stage	Number
$\varepsilon_{r}$	ENS	180	$\tau_{rms}$	SDS	25
$sum_{r}$	ENS	463	$mean_{r}$	ENS	402
$\sigma_{r}$	SDS	96	*k*	SDS	220
$\tau_{med}$	SRS	-	ske	SDS	96

**Table 4:** The UWB NLOS identification performance.

Scene	Method	Accuracy	${\mathrm{Recall}}_{\mathrm{LOS}}$	${\mathrm{Recall}}_{\mathrm{NLOS}}$	Scene	Method	Accuracy	${\mathrm{Recall}}_{\mathrm{LOS}}$	${\mathrm{Recall}}_{\mathrm{NLOS}}$
STA-1	Two-Step	93.32	92.82	93.63	STA-3	Two-Step	98.17	98.98	96.94
	K-NN [30]	77.11	74.81	81.61		K-NN	81.97	77.60	82.78
	LS-SVM [21]	83.00	89.90	81.70		LS-SVM	85.50	93.38	81.60
	CNN [24]	89.01	91.20	87.43		CNN	94.13	95.60	92.62
STA-2	Two-Step	93.44	94.18	92.91	STA-4	Two-Step	95.28	96.88	93.12
	K-NN	80.65	73.72	86.87		K-NN	75.48	73.56	79.89
	LS-SVM	84.00	92.85	83.20		LS-SVM	86.90	93.20	80.50
	CNN	90.51	93.60	88.72		CNN	92.37	93.03	89.09

**Table 5:** Error correction effect across different scenarios of I-NLOS (m).

		I-LOS				I-NLOS			
		Mean	STD	RMSE		Mean	STD	RMSE	
STA-1	Original	0.0566	0.2131	0.0454	STA-1	Original	1.2615	1.7035	2.9014
	Mitigated	0.0513	0.2085	0.0435		Mitigated	0.4850	0.6658	0.4432
STA-2	Original	0.0546	0.2028	0.0411	STA-2	Original	1.1315	1.6692	2.7859
	Mitigated	0.0476	0.1746	0.0305		Mitigated	0.2524	0.8736	0.7630
STA-3	Original	0.1571	0.4814	0.2319	STA-3	Original	2.6189	4.4700	5.9747
	Mitigated	0.0595	0.2244	0.0503		Mitigated	0.5988	1.4471	2.0936
STA-4	Original	0.1209	0.4095	0.1677	STA-4	Original	0.7854	1.9021	3.6174
	Mitigated	0.1010	0.3987	0.1532		Mitigated	0.2904	0.7557	0.5709

**Table 6:** Positioning performance of the algorithms in the three DYN experiments with different obstacles (m).

	DYN-1			DYN-2			DYN-3		
**Method**	**Mean**	**STD**	**RMSE**	**Mean**	**STD**	**RMSE**	**Mean**	**STD**	**RMSE**
Original	0.0953	0.1563	0.0244	0.2014	0.2166	0.0469	1.0329	1.9864	3.9425
Mitigated	0.0424	0.0774	0.0110	0.0834	0.0999	0.0260	0.5237	0.8001	1.6396
K-NN	0.0638	0.1036	0.0169	0.1418	0.1405	0.0301	0.7218	1.2940	2.2721
LS-SVM	0.0607	0.0946	0.0152	0.1225	0.1310	0.0325	0.6535	1.2264	2.1731
CNN	0.0551	0.0847	0.0139	0.1201	0.1207	0.0292	0.6287	1.0112	1.9799
LSTM-EKF [14]	0.0509	0.0907	0.0128	0.1016	0.1101	0.0285	0.6041	0.9239	1.8953

## Data Availability

Data sharing is not applicable due to the privacy policy.
